# Gliomas Infiltrating the Corpus Callosum: A Systematic Review of the Literature

**DOI:** 10.3390/cancers14102507

**Published:** 2022-05-19

**Authors:** Paolo Palmisciano, Gianluca Ferini, Gina Watanabe, Christian Ogasawara, Emal Lesha, Othman Bin-Alamer, Giuseppe E. Umana, Kenny Yu, Aaron A. Cohen-Gadol, Tarek Y. El Ahmadieh, Ali S. Haider

**Affiliations:** 1 Department of Neurosurgery, University of Cincinnati College of Medicine, Cincinnati, OH 45229, USA; 2 Department of Radiation Oncology, REM Radioterapia srl, 95029 Viagrande, Italy; gianluca.ferini@grupposamed.com; 3 John A. Burns School of Medicine, University of Hawai’i, Honolulu, HI 96813, USA; ginaw@hawaii.edu (G.W.); cogasawa@hawaii.edu (C.O.); 4 Department of Neurosurgery, University of Tennessee Health Science Center, Memphis, TN 38163, USA; elesha@uthsc.edu; 5 Department of Neurosurgery, University of Pittsburgh Medical Center, Pittsburgh, PA 15213, USA; binalameroa@upmc.edu; 6 Department of Neurosurgery, Trauma Center, Gamma Knife Center, Cannizzaro Hospital, 95126 Catania, Italy; umana.nch@gmail.com; 7 Department of Neurosurgery, Memorial Sloan Kettering Cancer Center, New York, NY 10065, USA; yuk2@mskcc.org (K.Y.); elahmadt@mskcc.org (T.Y.E.A.); 8 Department of Neurological Surgery, Indiana University School of Medicine, Indianapolis, IN 46202, USA; cohen@nsatlas.com; 9 Department of Neurosurgery, The University of Texas M.D. Anderson Cancer Center, Houston, TX 77030, USA; aalam@mdanderson.org

**Keywords:** butterfly glioma, corpus callosum, glioblastoma, neuro-oncology, survival

## Abstract

**Simple Summary:**

Gliomas infiltrating the corpus callosum (G-I-CC) may carry significant tumor burden by causing severe neurocognitive and functional impairments. The role of surgical resection has been widely debated over the years, as it has been correlated with significant survival improvement but may also predispose the patient to major post-operative complication risks. The aim of our systematic review was to comprehensively analyze the current literature on G-I-CC, describing clinical presentations, management strategies, outcomes, and prognoses. We found that most G-I-CC are IDH-wildtype grade-4 glioblastomas involving the corpus callosum genu and with bilateral lobe infiltration. In patients with high-grade G-I-CC, surgical resection, especially gross-total, led to significantly longer survival when coupled with post-surgery radiation and temozolomide. Rates of symptom improvement and complications did not significantly differ in preservation versus resection of tumor-infiltrated corpus callosum. Overall, maximally safe resection should be considered in patients with G-I-CC, co-adjuvated with intraoperative neuromonitoring and cortical mapping to further reduce complication risks.

**Abstract:**

Background: Gliomas infiltrating the corpus callosum (G-I-CC) majorly impact patient quality-of-life, but maximally safe tumor resection is challenging. We systematically reviewed the literature on G-I-CC. Methods: PubMed, EMBASE, Scopus, Web of Science, and Cochrane were searched following the PRISMA guidelines to include studies of patients with G-I-CC. Clinicopathological features, treatments, and outcomes were analyzed. Results: We included 52 studies comprising 683 patients. Most patients experienced headache (33%), cognitive decline (18.7%), and seizures (17.7%). Tumors mostly infiltrated the corpus callosum genu (44.2%) with bilateral extension (85.4%) into frontal (68.3%) or parietal (8.9%) lobes. Most G-I-CC were glioblastomas (84.5%) with IDH-wildtype (84.9%) and unmethylated MGMT promoter (53.5%). Resection (76.7%) was preferred over biopsy (23.3%), mostly gross-total (33.8%) and subtotal (32.5%). The tumor-infiltrated corpus callosum was resected in 57.8% of cases. Radiation was delivered in 65.8% of patients and temozolomide in 68.3%. Median follow-up was 12 months (range, 0.1–116). In total, 142 patients (31.8%) experienced post-surgical complications, including transient supplementary motor area syndrome (5.1%) and persistent motor deficits (4.3%) or abulia (2.5%). Post-treatment symptom improvement was reported in 42.9% of patients. No differences in rates of complications (*p* = 0.231) and symptom improvement (*p* = 0.375) were found in cases with resected versus preserved corpus callosum. Recurrences occurred in 40.9% of cases, with median progression-free survival of 9 months (0.1–72). Median overall survival was 10.7 months (range, 0.1–116), significantly longer in low-grade tumors (*p* = 0.013) and after resection (*p* < 0.001), especially gross-total (*p* = 0.041) in patients with high-grade tumors. Conclusions: G-I-CC show clinicopathological patterns comparable to other more frequent gliomas. Maximally safe resection significantly improves survival with low rates of persistent complications.

## 1. Introduction

Gliomas are the second most common primary tumors of the central nervous system (CNS), causing high morbidity and mortality burden worldwide [1]. Despite the promising advances in targeted therapy, oncolytic viral therapy, and immunotherapy, the favorable prognostic role of maximally safe tumor resection with adjuvant chemotherapy and radiation defines the current gold standard [2,3,4]. However, the infiltrative behavior of gliomas, theorized to be carried out via white-matter tracts and/or blood vessels, coupled with their frequent location in eloquent areas, poses major challenges in achieving gross-total resection while minimizing complications [5,6].

The corpus callosum (CC) represents the largest interhemispheric brain commissural tract, composed of dense myelinated white-matter fibers that connect two homologous cortical brain areas [7]. The CC may provide a feasible bridge for tumor cells to migrate into the contralateral hemisphere, as is often reported in gliomas arising from frontal or parietal lobes [8]. Sometimes referred to as “butterfly gliomas” due to their radiological appearance when symmetrically invading both hemispheres around the CC, gliomas infiltrating the corpus callosum (G-I-CC) are characterized as rare entities of complex management and poor prognosis [9,10]. Historically, the attempt to achieve maximally safe resection has been widely debated, as opposed to diagnostic biopsy followed by chemotherapy and radiation [11]. More recently, surgery has been preferred as it shows benefits in overall survival superior to biopsy, but it still comes with an increased risk of aggravating preexisting neurological deficits [10,12,13]. Novel intraoperative imaging and neuromonitoring techniques may serve as favorable adjuncts to improve surgical resection safety and extent, but are limited by strict requirements in costs and personnel training [14].

Due to the uncommon incidence of G-I-CC, most conclusions on the management of these lesions derive from case reports and small series with heterogeneous data on clinical outcomes [15,16]. In this systematic review, we comprehensively summarized clinical features, molecular alterations, management strategies, and their impact on survival in patients with G-I-CC.

## 2. Materials and Methods

### 2.1. Literature Search

A systematic review was conducted following the Preferred Reporting Items for Systematic Reviews and Meta-Analyses (PRISMA) guidelines [17] and registered to PROSPERO (ID: 324555). PubMed, EMBASE, Scopus, Web of Science, and Cochrane were searched from database inception to 27 March 2022, using the combination of Boolean operators “OR” and “AND” and search terms: “corpus callosum”, “butterfly”, “glioblastoma*”, and “gliom*”. Studies were uploaded to Mendeley, and duplicates were deleted.

### 2.2. Study Selection

Predetermined inclusion and exclusion criteria were set. Studies were included if they: (1) involved ≥1 patients with histologically confirmed gliomas involving the corpus callosum as explicitly mentioned by the authors or extracted from radiological images; (2) reported data on clinical features, treatments, and outcomes; (3) were written in English. Studies were excluded if they were: (1) reviews, conference abstracts, animal studies, or autopsy reports; (2) studies with unclear distinction between patients with G-I-CC and without; (3) studies lacking data on ≥2 of the following: clinical characteristics, histomolecular alterations, treatments, and/or outcomes. In the case of studies with overlapping cohorts, only the ones with the longest follow-up were included.

Two independent reviewers (G.W. and C.O.) screened titles and abstracts of all collected articles, and then assessed full-texts of those meeting the inclusion criteria. A third reviewer (P.P.) resolved any conflict. Eligible articles were included and references were scrutinized to retrieve additional relevant studies.

### 2.3. Data Extraction

Data were extracted by two reviewers (G.W. and C.O.) and confirmed by one additional reviewer (P.P.). Missing data were not reported by the authors. Extracted data included: authors, year, sample size, age, gender, symptoms, tumor location in the CC and laterality, hemisphere extension, WHO grade and type valid at the time of study publication, histomolecular patterns, extent-of-surgery, CC resection, complications, post-surgery treatments, symptom response, recurrence, progression-free survival (PFS), overall survival (OS), and survival status. Extent-of-resection was defined as “gross-total resection” for 90–100% tumor resection, “subtotal resection” for 80–90%, and “partial resection” for <80%. Surgical complications were divided into “transient”, if self-resolved at later follow-ups, and “persistent”, if untreatable or requiring additional operations. Symptom responses were assessed at last available follow-up.

### 2.4. Data Synthesis and Quality Assessment

Primary outcomes of interest were clinicopathological characteristics, management, and outcomes in patients with G-I-CC. For each article, two independent reviewers (P.P. and C.O.) appraised level of evidence using the 2011 Oxford Centre For Evidence-Based Medicine guidelines, and risk of bias using the Joanna Briggs Institute checklists for case reports and case series [18,19]. The overall risk of bias for this review was determined by considering the aggregate risk of bias of all included studies. A study-level meta-analysis was precluded because the included studies had levels IV–V of evidence and hazard ratios could not be deducted. A patient-level meta-analysis was conducted using individual patient-level data [20].

### 2.5. Statistical Analysis

SPSS V.25 (IBM Corp, Armonk, NY, USA) was operated and bilateral *p*-values < 0.05 were considered significant for all tests. Continuous variables are summarized as medians and ranges, and categorical variables as frequencies and percentages. Rates of post-treatment outcomes and complications based on CC resection were compared using χ^2^ and Fisher exact tests. Time intervals between surgery and tumor recurrence (PFS curve) or death (OS curve) were estimated with the Kaplan–Meier method, and survival analyses were conducted with the log-rank test.

## 3. Results

### 3.1. Study Selection

**Figure 1:**
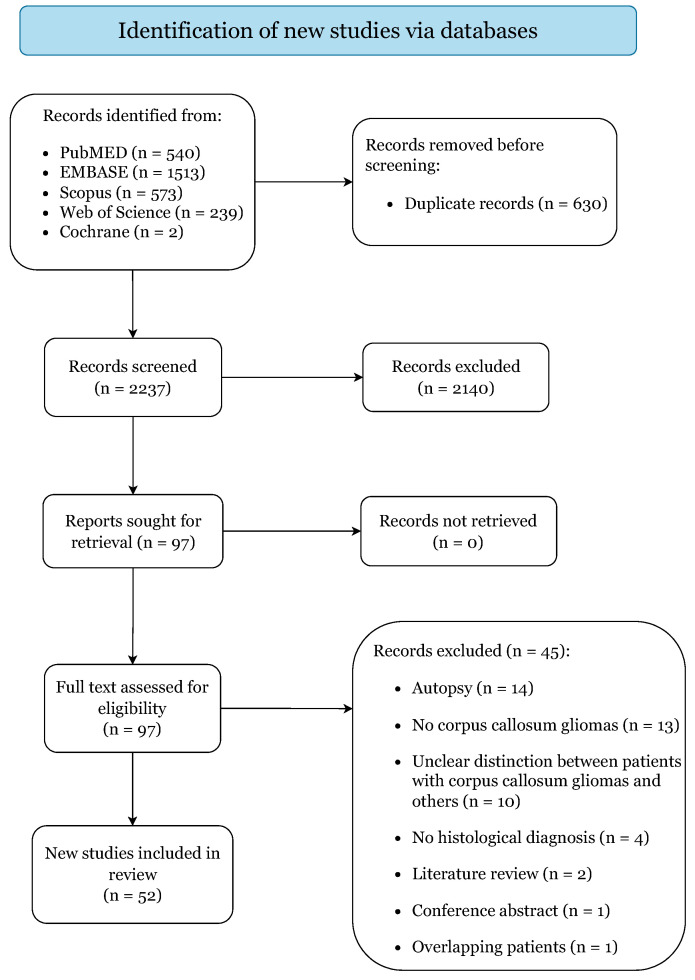
PRISMA 2020 flow diagram.

Figure 1 illustrates the study selection process. The initial search yielded 2867 citations (PubMed: 540; EMBASE: 1513; Scopus: 573; Web of Science: 239; Cochrane: 2). A total of 19 case series and 33 case reports were included, categorized as level IV and V of evidence (Supplementary File S1) [9,10,12,13,14,21,22,23,24,25,26,27,28,29,30,31,32,33,34,35,36,37,38,39,40,41,42,43,44,45,46,47,48,49,50,51,52,53,54,55,56,57,58,59,60,61,62,63,64,65,66,67]. Critical assessment returned low risk of bias for all included studies, predisposing this review to a low overall risk of bias (Supplementary File S2) [9,10,12,13,14,21,22,23,24,25,26,27,28,29,30,31,32,33,34,35,36,37,38,39,40,41,42,43,44,45,46,47,48,49,50,51,52,53,54,55,56,57,58,59,60,61,62,63,64,65,66,67].

### 3.2. Clinicoradiological Characteristics and Histomolecular Patterns

Table 1 summarizes the clinical characteristics of all 683 included patients. Median age was 54 years (range, 0.5–83) with a male prevalence (56.1%). Patients experienced various degrees of debilitating symptoms, most commonly headache (33%), confusion or cognitive decline (18.7%), seizures (17.7%), motor deficits (13.5%), and memory loss (13.1%). Altered consciousness was reported in 11 cases (2.2%) and personality changes in 9 (1.8%). Bouali et al. [50] treated one infant with CC gliosarcoma presenting with hydrocephalus, macrocephalia, and failure to thrive. In five patients (1%), asymptomatic G-I-CC were diagnosed incidentally at oncological follow-up imaging. Tumors most frequently invaded the genu of the CC (44.2%) and extended bilaterally (i.e., “butterfly glioma”) (85.4%) into frontal (68.3%) or parietal (8.9%) lobes. Less commonly, G-I-CC unilaterally infiltrated the adjacent brain hemispheres (14.4%). Azriel et al. [45] described one patient with a glioblastoma located only in the CC, showing no hemisphere infiltration. Grade-4 glioblastomas comprised the most common lesions (84.5%), followed by grade-2 astrocytomas (9.7%). Three cases (0.4%) of grade-4 gliosarcoma [50,51,62] and one case each (0.1%) of grade-1 ganglioglioma [55], grade-1 pilocytic astrocytoma [59], and grade-1 subependymoma [67] were reported. Among cases with available histomolecular data, most tumors showed IDH-wildtype (84.9%), unmethylated MGMT promoters (53.5%), non-amplified EGFR (61.6%), mutated p53 (53.5%), non-mutated PTEN (46.3%), and normal ATRX (100%). La Rocca et al. [47] reported one case of an H3 K27-altered butterfly splenium glioblastoma.

### 3.3. Management Strategies

Treatment strategies are reported in Table 2. Tumor biopsy was obtained in 159 patients (23.3%), while resection was pursued in 524 (76.7%): gross-total (33.8%), subtotal (32.5%), or partial (10.4%). The tumor-infiltrated CC was resected in 395 cases (57.8%). Surgeries were mostly performed with open interhemispheric transcallosal or trans-sulcal approaches, often using intraoperative adjuncts including awake cortical/subcortical mapping [9,37], ultrasound [9,10,13], MRI [13,14], and/or sensorimotor neuromonitoring [10,13,14]. Dadario et al. [62] described their endoscopic-assisted interhemispheric trans-sulcal and transcortical techniques in 70 butterfly G-I-CC. Among patients with available data, 443 (73.6%) received post-surgery treatments, including radiotherapy (65.8%) and chemotherapy (68.8%), mostly temozolomide (68.3%). In early series, two patients (0.3%) were treated with procarbazine, lomustine, and vincristine [21], and one patient (0.2%) with intrathecal methotrexate [31].

### 3.4. Outcomes, Complications, and Survival

**Figure 2:**
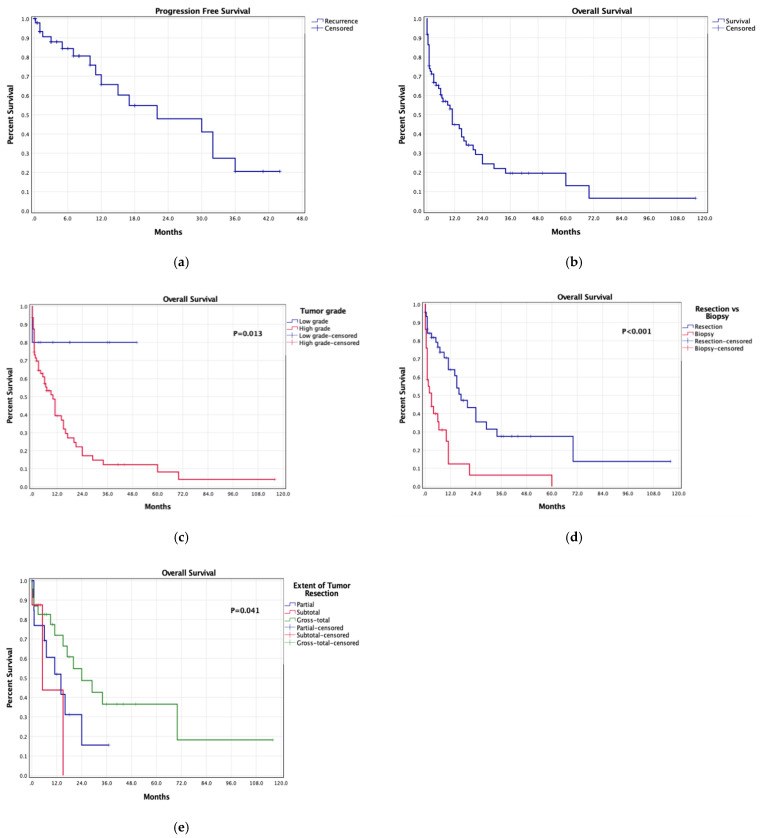
Kaplan–Meier survival curves: (**a**) pooled progression-free survival, (**b**) pooled overall survival, (**c**) overall survival based on tumor grade, (**d**) overall survival based on resection versus biopsy in patients with high-grade G-I-CC receiving adjuvant treatments, (**e**) overall survival based on the extent of tumor resection in patients with high-grade G-I-CC receiving adjuvant treatments.

Patients were followed-up for a median of 12 months (range, 0.1–116) (Table 2). A total of 142 patients (31.8%) experienced post-surgical complications, which were transient in 47 (10.5%), mostly supplementary motor area syndrome (SMAS, 5.1%) and motor deficits (2.2%), and persistent in 95 (21.3%), mostly motor deficits (4.3%), aphasia (2.7%), and abulia (2.5%). Post-operative persistent memory loss (1.6%) and cognitive decline (0.7%) were also reported. No significant differences in complication rates were noted between patients with resected-tumor-infiltrated CC and patients without (*p* = 0.231). Post-treatment symptom improvement was described in 42.9% of patients with available data, with no significant differences between CC resection and preservation (*p* = 0.375). Tumor recurrences were reported in 40.9% of patients with available data, with median PFS of 9 months (range, 0.1–72) in patients with high-grade G-I-CC (Figure 2). A total of 508 patients died at last follow-up (74.4%), showing a median OS of 10.7 months (0.1–116). Survival was significantly longer in low-grade (grade-1 and grade-2) G-I-CC than high-grade (grade-3 and grade-4) G-I-CC (*p* = 0.013). In patients with high-grade G-I-CC receiving post-surgery chemotherapy and/or radiation, survival was significantly longer after resection than biopsy (*p* < 0.001), and after gross-total than subtotal or partial resection (*p* = 0.041).

## 4. Discussion

Gliomas infiltrating the CC are uncommon yet challenging entities that significantly affect patient quality-of-life. While maximally safe resection remains the gold standard for providing symptom and survival improvement, complications related to CC resection need to be considered. In this review, we provided a comprehensive summary of the current literature on G-I-CC, showing that CC resection had no significant impact on post-surgery complications and symptom response, but tumor resection, especially gross-total, led to a significant increase in overall survival when compared to biopsy alone.

The CC regulates the functional connectivity and integration between the two cerebral hemispheres in transferring sensory, language, motor, and high-order functions [68]. Pathology disrupting the white-matter fibers alters the neural activity of functional networks and may result in major neurological impairment. Deemed to originate from subventricular pluripotent progenitor cells, glioma cells have been shown to preferentially migrate and infiltrate the healthy brain tissue along with the white-matter tracts [69]. As found in this review, progressive tumor growth within the CC may lead to a wide range of symptoms secondary to mass-effect and altered white-matter networks connectivity, induced by either direct tumor infiltration or surrounding edema. In addition to the increased intracranial pressure with severe headache, epilepsy and high-order function disorders, including cognitive decline, memory loss, and personality change, were frequently reported across our included studies [9,37,62]. Some presenting symptoms may be strictly related to the tumor’s involvement of frontal, parietal, or temporo-occipital lobes, with a smaller role determined by CC infiltration. Only the early study of Chaichana et al. [10] in 2014 compared the clinical presentation of butterfly and non-butterfly glioblastomas, reporting significant higher incidence of seizures (*p* < 0.001), language deficits (*p* = 0.05), and confusion (*p* < 0.001) in non-butterfly glioblastomas. Most recent studies noted the occurrence of specific high-function disorders in patients with G-I-CC, especially personality and/or behavioral changes. Although the recent literature has focused more on identifying the role of CC in high-function networks, no recent study compared the clinical presentations of gliomas with versus without CC involvement for high-function activities, limiting a clear understanding on how the CC invasion and disruption of white-matter tracts may uniquely affect patients’ well-being on a daily basis. Hence, our pooled findings from the literature highlight the importance of promptly diagnosing CC infiltration and devising appropriate management strategies to limit progressive deterioration of patient’s social interactions and functional autonomy, but further studies are necessary to elucidate and quantify the impact of G-I-CC compared to non-CC infiltrating gliomas.

G-I-CC may show variable involvement of the CC with different infiltrating radiological patterns along the white-matter fibers [34,44]. Most of our pooled G-I-CC involved the genu of the corpus callosum and bilaterally infiltrated the adjacent frontal lobes, while unilaterally infiltrating tumors were less common. This likely derived from the superior prevalence of highly invasive glioblastomas, which majorly disrupt white-matter interhemispheric networks with rapid neurological impairment, and from the surgeon’s hesitancy to operate on the more challenging G-I-CC involving the splenium [13]. Dayani et al. [44] found significantly longer survival in unilateral G-I-CC, which may suggest the impact of underlying molecular differences between bilateral and unilateral G-I-CC in determining distinct outcomes. In regards to butterfly G-I-CC, lesions showed symmetric or asymmetric lateralization patterns, with larger resection rates for asymmetric tumors reported by Dziurzynski et al. [34]. While conventional imaging can detect late CC infiltration after significant white-matter damage and brain-barrier disruption, it is less valuable at early tumor stages. Contrarily, Mohan et al. [70] effectively quantified “occult” early CC infiltration using diffusion tensor imaging techniques, which can serve as valuable prognostic indicators for G-I-CC.

In line with the current literature on gliomas, the majority of our pooled cases were diagnosed with grade-4 glioblastoma, unsurprisingly related with significantly shorter OS than low-grade G-I-CC [1,71]. CC infiltration has been demonstrated as an independent prognostic factor of worse OS in glioblastoma patients [10,42,72], further confirmed by the fact that our pooled median OS in G-I-CC (10.7 months) was lower than the pooled median OS in non-CC glioblastomas (13.2 months) [13]. It is reasonable to hypothesize the presence of underlying molecular alterations responsible for the higher aggressiveness and poorer prognosis of G-I-CC compared to other gliomas, as shown by the large prevalence of IDH-wildtype gliomas (84.9%) within our pooled cohort. Although the IDH mutation status defines distinct subsets of high-grade gliomas, especially grade-4 glioblastomas, Dadario et al. [62] found no significant survival differences between IDH-mutant (*n* = 33) and IDH-wildtype (*n* = 11) (*p* = 0.64). However, granular patient-level data on IDH mutation status were lacking across all included studies, preventing the characterization of G-I-CC clinical presentation and prognosis based on distinct molecular patterns. Future studies adopting the most recent WHO CNS tumor classification [73] should better describe and characterize the differences across G-I-CC on a molecular basis.

Shen et al. [74] and Cui et al. [75] found that mutations of the platelet-derived growth factor receptor alpha (PDGFRA) gene were significantly correlated with CC glioma infiltration and worse OS. PDGFRA is known to participate in gliomagenesis by transducing multiple downstream proliferative signals, and seems to also be involved in glioma invasion into dense white-matter fibers, as confirmed by studies on diffuse intrinsic pontine gliomas [76]. Since no reports of PDGFRA status were available across our included studies, future research should be conducted in large patient cohorts to validate or disprove the prognostic role of PDGFRA alterations in G-I-CC. Some cases of H3 K27-altered G-I-CC have been also reported [47], but the role of routine assessment for H3 K27 mutations in G-I-CC remains unclear as no cases of H3 K27-altered tumors were detected in a single-institution retrospective series of 49 G-I-CC [77]. Of note, Mistry et al. [41] suggested that G-I-CC’s worse prognosis is likely related to the tumor’s contact with the ventriculo-subventricular and/or granular zones (i.e., neurogenic zones harboring neural stem cells) rather than with the CC itself, but their findings have yet to be externally confirmed.

Historically, G-I-CC were deemed “inoperable” owing to the potentially severe post-resection complications, with early series preferring diagnostic biopsy to establish palliative care [21,61,65]. The advent of superior imaging techniques and intraoperative adjuncts, coupled with better knowledge of brain connectomics, has increased the surgical inclination toward G-I-CC resection, similarly to other gliomas [37,71,78,79]. Given its known association with improved survival, maximally safe tumor resection, when feasible, also represents the primary treatment for G-I-CC, further enabling patients to complete post-surgery radiation and chemotherapy protocols [34]. Surgeries are mostly performed with interhemispheric trans-sulcal and transcortical approaches, often assisted with intraoperative mapping and neuromonitoring for neurocognitive function preservation and/or intraoperative imaging tools for better tumor localization [9,10]. Boaro et al. [13] and Cui et al. [14] demonstrated that multimodal G-I-CC resection significantly correlates with higher rates of extended resection and lower rates of complications. Burks et al. [37] described their cingulum-sparing surgical technique, which involves awake subcortical anterior G-I-CC resection with high-order attention tasks to avoid disruption of the cingulum and default mode network (DMN). Using this awake monitoring technique, the authors preserved the functional DMN (i.e., the white-matter network involved in high-order attention) and noted significantly lower rates of post-surgery abulia/akinesia but no OS differences than their conventional surgical technique. In their cohort, Forster et al. [53] found no cases of abulia or post-operative callosal disconnection syndrome with both cingulum-resection and cingulum-sparing techniques, but noted higher rates of post-operative transient supplementary motor area syndrome after resection of tumors within the corpus callosum. More recently, Dadario et al. [62] achieved ≥95% G-I-CC resection with minimal complication rates using their endoscope-assisted transcortical or interhemispheric trans-sulcal approach, which offered angled surgical views, allowing optimal instrument maneuvering around complex anatomical structures.

Maximal resection of high-grade G-I-CC combined with adjuvant radiation and/or chemotherapy was correlated with significantly longer OS compared to tumor biopsy (*p* < 0.001) or partial resection (*p* = 0.041). Although the early study of Chaichana et al. [10] in 2014 noted a significantly shorter median survival in butterfly glioblastomas (*n* = 40) compared to non-butterfly glioblastomas (*n* = 336) (*p* < 0.001), mostly treated before the introduction of Stupp protocols, the heterogeneity between the two cohorts in terms of size and treatment strategies (i.e., higher GTR rates in non-butterfly glioblastomas) largely limited the actual quantification of the impact of CC-invasion on patients’ prognosis. No further comparative studies analyzed the prognostic role of glioma’s CC-invasion on patient’s survival, impeding the conduction of a retrospective comparative literature review on gliomas with CC infiltration versus gliomas without CC infiltration. Future retrospective and prospective studies are warranted to compare matching cohorts of patients with G-I-CC and non-CC infiltrating gliomas to define the prognostic impact of CC-invasion and other related factors (e.g., tumor type and size, CC location, treatment strategies).

Management strategies should also focus on improving patient quality-of-life by relieving symptoms while minimizing complication risks. We assume that the moderate rates of symptom improvement noted in our pooled cohort (42.9%) were likely related to irreversible tumor-induced disruption of white-matter networks. Similarly, the occurrence of post-surgery complications likely derived from the surgical manipulation of white-matter tracts with post-operative edema formation, often leading to motor deficits and cognitive decline, especially in patients with splenium G-I-CC [13,62]. Forster et al. [53] suggested three prognostic factors likely related to their low incidence of post-surgery complications, including: (1) selection of patients with favorable preoperative performance status scores; (2) tumors mainly involving the frontal lobe and genu/cingulum of the CC; (3) surgical resection performed with intraoperative awake subcortical mapping and neuromonitoring. The authors also analyzed the rates of neurocognitive impairment before and after tumor resection. In contrast to Ng et al. [80], who reported early post-surgery cognitive improvement (except for executive function) after resection of lobar gliomas, Forster et al. [53] noted early post-operative deterioration in all cognitive domains with significant improvement at later long-term follow-up times. The only permanent deficits involved functions critically depending on interhemispheric transfer, such as visual memory, altered with lesions or surgeries involving the genu or cingulum, and bimanual coordination with tactile recognition, impaired after disruption of the CC’s posterior body and splenium. Of note, owing to the frequent frontal and/or parietal lobe involvement of G-I-CC, we observe that some post-surgical complications may be related to lobar damage during the tumor’s resection [81]. However, currently available studies were not able to differentiate post-operative complications related to CC resection versus those related to frontal/parietal lobe resections, owing to the difficulty of characterizing such iatrogenic outcomes in a retrospective fashion. Future studies, designed in a prospective comparative cohort fashion, may be able to differentiate and quantify the impact of surgical disruption of distinct white-matter tracts during G-I-CC surgery using diffusion tensor techniques and/or machine-learning-based connectomics algorithms currently available for preoperative planning of tumor resection [82].

In this review, no significant differences in pooled rates of outcomes and complications were noted after resection of tumor-infiltrated CC. This probably stems from the structural and functional reorganization of interhemispheric CC connectivity induced by progressive tumor growth and disruption of white-matter tracts [68]. In addition, some studies noted higher rates of G-I-CC recurrence in patients with non-resected tumor-infiltrated CC, confirming the role of gross-total resection in obtaining prolonged rates of tumor control [9,13]. Hence, the tumor-infiltrated CC may be safely resected during G-I-CC surgery, but preoperative advanced imaging planning and intraoperative monitoring of high-order neurocognitive functions should be appropriately considered on a case-by-case basis.

### Limitations

Our review has some limitations. All pooled studies were retrospective and were likely subjected to patient selection and data recall biases. These studies also covered a 73-year time period characterized by major changes in histopathological grading and adjuvant treatments, which may have introduced confounding variables into our analyses. Publication bias needs to be considered, favoring series from centers experienced in surgery of deep-seated tumors, and with possible reporting biases presenting patients with overall positive post-operative outcomes. The assessment of post-treatment clinical improvement was subjective in most studies. As with all retrospective extent-of-resection survival analyses, there is significant selection bias in tumor anatomy and safety of removal. In addition, data on molecular alterations, in particular IDH mutation status, were not available on a patient-level basis, which prevented us from performing separate survival analyses for IDH-mutant gliomas and IDH-wildtype gliomas. Due to lack of granular data, we could not comprehensively analyze objective post-operative changes in patients’ performance status, nor the impact of tumor size on clinical outcomes, nor the impact of CC resection on tumor recurrence, nor the impact of CC resection compared to frontal/parietal lobe damage on post-surgical complications. Finally, in view of the high heterogeneity within the current literature on lobar non-CC infiltrating gliomas, coupled with the limited availability of studies reporting G-I-CC, we note that a retrospective systematic review/meta-analysis comparing the two entities is not currently pursuable. Yet, as CC-infiltration is of high clinical relevance in the management of patients with gliomas, especially in terms of patient’s prognosis and quality-of-life, future original cohort studies should strictly analyze the unique impact of CC-invasion on survival outcomes to better devise treatment strategies for these patients.

## 5. Conclusions

Gliomas infiltrating the CC are uncommon yet challenging neoplasms, with a major impact on patient cognitive and functional status. Maximally safe tumor resection combined with adjuvant treatment should be preferred but weighted to surgical risks. Novel intraoperative adjuncts and surgical techniques may offer higher tumor resection rates while minimizing complication risks. G-I-CC appear to be related to more aggressive underlying molecular patterns. Future studies should evaluate the role of PDGFRA and H3 K27 alterations in predisposition to CC invasion by glioma cells and worse clinical outcomes.

## Figures and Tables

**Table 1:** Summary of clinicoradiological and histomolecular features of all pooled patients.

Characteristics	Value
Cohort size (no.)	683
Demographics	
Age (years), median (range)	54 (0.5–83)
Gender (male)	383 (56.1%)
Presenting symptoms (*n* = 503)	No. (%)
Headache	166 (33%)
Confusion/cognitive decline	94 (18.7%)
Seizure	89 (0.2%)
Motor deficit	68 (13.5%)
Memory loss	66 (13.1%)
Nausea and vomit	43 (8.5%)
Speech disorder	38 (7.6%)
Vision deficit	32 (6.4%)
Sensory deficit	24 (4.8%)
Altered consciousness	11 (2.2%)
Ataxia	11 (2.2%)
Behavior/personality change	9 (1.8%)
Cranial nerve neuropathies	3 (0.6%)
Intracranial hemorrhage	1 (0.2%)
Macrocephalia and failure to thrive	1 (0.2%)
No symptoms	5 (1%)
Location in corpus callosum (*n* = 486)	No. (%)
Genu	215 (44.2%)
Genu/body	89 (18.3%)
Body	65 (13.4%)
Body/splenium	27 (5.6%)
Splenium	90 (18.5%)
Laterality (*n* = 576)	No. (%)
Butterfly (bilateral)	492 (85.4%)
Unilateral	83 (13.4%)
Limited to the corpus callosum	1 (0.2%)
Hemisphere infiltration (*n* = 347)	No. (%)
Frontal lobe	237 (68.3%)
Parietal lobe	31 (8.9%)
Frontoparietal lobe	26 (7.5%)
Frontotemporal lobe	24 (6.9%)
Parietooccipital lobe	17 (4.9%)
Parietotemporal lobe	11 (3.2%)
Limited to the corpus callosum	1 (0.3%)
WHO grade and type	No. (%)
1-Ganglioglioma	1 (0.1%)
1-Pilocytic astrocytoma	1 (0.1%)
1-Subependymoma	1 (0.1%)
2-Astrocytoma	66 (9.7%)
2-Oligodendroglioma	4 (0.6%)
3-Anaplastic astrocytoma	18 (2.6%)
3-Anaplastic oligoastrocytoma	7 (1%)
3-Anaplastic oligodendroglioma	5 (0.7%)
4-Glioblastoma	577 (84.5%)
4-Gliosarcoma	3 (0.4%)
Molecular patterns (*n* = 392)	No. (%)
IDH-1 mutated	52/344 (15.1%)
IDH-1 wildtype	292/344 (84.9%)
MGMT promoter methylated	106/228 (46.5%)
MGMT promoter unmethylated	122/228 (53.5%)
EGFR amplified	53/138 (38.4%)
P53 mutated	60/96 (53.5%)
PTEN mutated	25/54 (46.3%)
ATRX normal	30/30 (100%)
H3 K27-altered	1/1 (100%)

**Table 2:** Summary of treatment strategies and outcomes of all pooled patients.

Characteristics	Value
Surgical Management	No. (%)
Biopsy	159 (23.3%)
Tumor resection	524 (76.7%)
Gross-total (90–100%)	231 (33.8%)
Subtotal (80–90%)	222 (32.5%)
Partial (<80%)	71 (10.4%)
Resection of corpus callosum	395 (57.8%)
Post-surgery treatments (*n* = 602)	No. (%)
Radiotherapy	396 (65.8%)
Chemotherapy	414 (68.8%)
Temozolomide	411 (68.3%)
Procarbazine + lomustine + vincristine	2 (0.3%)
Intrathecal methotrexate	1 (0.2%)
Surgical complications (*n* = 447)	No. (%)
Transient	47 (10.5%)
Supplementary motor area syndrome	23 (5.1%)
Motor deficit	10 (2.2%)
Abulia	5 (1.1%)
Sensory deficit	4 (0.9%)
Confusion	3 (0.7%)
Dysphasia	3 (0.7%)
Vision deficit	3 (0.7%)
Neglect	2 (0.4%)
Persistent	95 (21.3%)
Motor deficit	19 (4.3%)
Aphasia	12 (2.7%)
Abulia	11 (2.5%)
Hemorrhage	10 (2.2%)
Hydrocephalus	10 (2.2%)
Skin infection	9 (2%)
Infarct	8 (1.8%)
Memory loss	7 (1.6%)
Seizure	6 (1.3%)
Meningitis	5 (1.1%)
Vision deficits	4 (0.9%)
Cognitive decline	3 (0.7%)
Cranial nerve neuropathies	2 (0.4%)
Neglect	1 (0.2%)
Symptom improvement (*n* = 198)	85 (42.9%)
Recurrence (*n* = 328)	134 (40.9%)
Survival	
Follow-up (months), median (range)	12 (0.1–116)
Progression-free survival (months), median (range)	9 (0.1–72)
Overall survival (months), median (range)	10.7 (0.1–116)
Status	No. (%)
Alive	175 (25.6%)
Dead	508 (74.4%)

## Supplementary Materials

The following supporting information can be downloaded at: https://www.mdpi.com/article/10.3390/cancers14102507/s1, Supplementary File S1: Overview of all included studies; Supplementary File S2: Risk of bias assessments for all included studies.
