# Supplementary material for: Gliomas Infiltrating the Corpus Callosum: A Systematic Review of the Literature
Source: Cancers (Basel). 2022 May 19;14(10):2507. doi: 10.3390/cancers14102507 (PMC9139932; doi:10.3390/cancers14102507)
Supplement: Supplementary file 1 [file cancers-14-02507-s001.zip › Supplementary File 1 - Overview of all included studies.pdf]

Supplementary File S1. Overview of all included studies.

	Authors - Year	Study Design - Level of evidence	Patients No./ male	Age years median (range)	Location in Corpus Callosum No. patients (percentage)	Unilateral or Butterfly Invasion No. patients (percentage)	WHO Grade – Type No. patients (percentage)	Extent of Tumor Resection No. patients (percentage)	Resection of Corpus Callosum No. patients (percentage)	Surgical Complications No. patients (percentage)	Radiotherapy No. patients (percentage)	Chemotherapy No. patients (percentage)	Symptom Improve No. patients (percentage)	Recurrence No. patients (percentage)	Progression Free Survival months median (range)	Overall Survival months median (range)	Alive No. patients (percentage)
1	**Cabot et al. – 1949 [61]**	Case report V	1 / 1 (100%)	62	Body	Butterfly	4-GBM	Biopsy	No	0 (0%)	0 (0%)	0 (0%)	0 (0%)	0 (0%)	N/A	0.1	0 (0%)
2	**Anderson et al. – 1970 [64]**	Case report V	1 / 1 (100%)	6	Body	Unilateral	3-AA	Biopsy	No	0 (0%)	1 (100%)	0 (0%)	1 (100%)	0 (0%)	N/A	5	1 (100%)
3	**Zant et al. – 1982 [65]**	Case report V	1 / 0 (0%)	53	Splenium	Butterfly	4-GBM	Biopsy	No	0 (0%)	1 (100%)	0 (0%)	1 (100%)	0 (0%)	N/A	4	0 (0%)
4	**Kato et al. – 1990 [66]**	Case report V	1 / 1 (100%)	66	Genu	Butterfly	4-GBM	PR	No	Persistent	0 (0%)	0 (0%)	0 (0%)	0 (0%)	N/A	1	0 (0%)
5	**Lindboe et al. – 1992 [67]**	Case report V	1 / 1 (100%)	63	Genu	Butterfly	1-SE	STR	No	Persistent	0 (0%)	0 (0%)	0 (0%)	0 (0%)	N/A	0.1	0 (0%)
6	**Bauman et al. – 1998 [21]**	Case series IV	2 / 2 (100%)	53 (42-64)	Genu 2 (100%)	Butterfly 2 (100%)	3-AA 1 (50%)<br>4-GBM 1 (50%)	Biopsy 2 (100%)	No 2 (100%)	0 (0%)	2 (100%)	2 (100%)	0 (0%)	0 (0%)	N/A	1.5	0 (0%)
7	**Ideguchi et al. – 1998 [22]**	Case report V	1 / 1 (100%)	72	Splenium	Butterfly	3-AA	STR	No	Persistent	0 (0%)	0 (0%)	1 (100%)	0 (0%)	N/A	0.5	0 (0%)
8	**Bampoe et al. – 1999 [23]**	Case report V	1 / 1 (100%)	52	Genu/Body	Butterfly	3-AA	STR	No	Transient	1 (100%)	0 (0%)	1 (100%)	0 (0%)	N/A	4	1 (100%)
9	**Duffau et al. – 2004 [9]**	Case series IV	32 / 15 (46.9%)	36 (22-55)	Body 12 (37.5%)<br>Genu 10 (31.2%)<br>Genu/Body 5 (15.6%)	Unilateral 32 (100%)	2-A 32 (100%)	STR 18 (56.2%)<br>GTR 9 (28.1%)<br>PR 5 (15.6%)	Yes 32 (100%)	Transient 25 (78.1%)<br>Persistent 1 (3.1%)	0 (0%)	0 (0%)	31 (96.9%)	2 (6.2%)	42 (12-72)	36 (9-84)	32 (100%)
10	**Price et al. – 2004 [24]**	Case report V	1 / 0 (0%)	59	Genu	Butterfly	4-GBM	STR	No	0 (0%)	0 (0%)	0 (0%)	1 (100%)	1 (100%)	1.5	2	1 (100%)
11	**Pallud et al – 2005 [25]**	Case report V	1 / 0 (0%)	25	Genu/Body	Butterfly	3-AODG	PR	No	0 (0%)	1 (100%)	1 (100%)	1 (100%)	0 (0%)	N/A	6	1 (100%)
12	**Voloschin et al. – 2005 [26]**	Case report V	1 / 0 (0%)	38	Genu	Unilateral	3-AODG	GTR	No	0 (0%)	0 (0%)	1 (100%)	1 (100%)	0 (0%)	N/A	7	1 (100%)
13	**Zakrzewska et al. – 2007 [27]**	Case report V	1 / 1 (100%)	16	Body	Butterfly	4-GBM	PR	No	0 (0%)	0 (0%)	0 (0%)	0 (0%)	1 (100%)	1	1.1	0 (0%)
14	**Tsutsumi et al. – 2008 [28]**	Case report V	1 / 0 (0%)	11	Genu	Butterfly	4-GBM	STR	No	0 (0%)	0 (0%)	1 (100%)	0 (0%)	1 (100%)	0.2	5.1	0 (0%)
15	**Agrawal et al. – 2009 [29]**	Case report V	1 / 1 (100%)	70	Splenium	Butterfly	4-GBM	Biopsy	No	0 (0%)	0 (0%)	0 (0%)	0 (0%)	0 (0%)	N/A	1	0 (0%)
16	**Steiger et al. – 2009 [30]**	Case series IV	11 / 6 (54.5%)	37 (20-65)	Genu 11 (100%)	Unilateral 11 (100%)	2-A 3 (27.3%)<br>2-ODG 1 (9.1%)<br>3-AOA 7 (63.6%)	GTR 8 (72.7%)<br>PR 3 (27.3%)	Yes 8 (72.7%)<br>No 3 (27.3%)	Persistent 2 (18.1%)	7 (63.6%)	0 (0%)	9 (81.8%)	6 (54.5%)	26 (5-32)	32 (12-116)	8 (72.7%)
17	**Kumar et al. – 2010 [31]**	Case report V	1 / 1 (100%)	16	Splenium	Butterfly	3-AA	GTR	Yes	Persistent	0 (0%)	1 (100%)	0 (0%)	0 (0%)	N/A	3	0 (0%)
18	**Monaco et al. – 2011 [32]**	Case report V	1 / 0 (0%)	52	Genu/Body	Butterfly	2-ODG	PR	Yes	0 (0%)	1 (100%)	0 (0%)	1 (100%)	0 (0%)	N/A	18	1 (100%)
19	**Witoonpanich et al. – 2011 [33]**	Case report V	1 / 1 (100%)	64	Body/Splenium	Butterfly	4-GBM	Biopsy	No	0 (0%)	0 (0%)	0 (0%)	0 (0%)	0 (0%)	N/A	3	0 (0%)
20	**Dziurzynskiet al. – 2012 [34]**	Case series IV	23 / 11 (47.8%)	59 (30-77)	Genu 13 (50%)<br>Body 6 (26.1%)<br>Splenium 3 (13%)<br>Genu/Body 1 (4.3%)	Butterfly 23 (100%)	4-GBM 23 (100%)	Biopsy 12 (52.2%)<br>GTR 6 (26.1%)<br>PR 5 (21.7%)	No 17 (73.9%)<br>Yes 6 (26.1%)	N/A	12 (52.2%)	9 (15.3%)	N/A	N/A	N/A	6 (0.1-34)	0 (0%)
21	**Chaichana et al. – 2014 [10]**	Case series IV	48 / 25 (52.1%)	60 (40-72)	N/A	Butterfly 48 (100%)	4-GBM 48 (100%)	Biopsy 19 (39.6%)<br>STR 19 (39.6%)<br>PR 10 (20.8%)	No 48 (100%)	Persistent 9 (18.8%)	32 (66.7%)	29 (60.4%)	N/A	N/A	N/A	6 (0.1-36)	0 (0%)
22	**Lee et al. – 2014 [35]**	Case report V	1 / 0 (0%)	30	Genu	Unilateral	3-AA	GTR	No	0 (0%)	1 (100%)	0 (0%)	1 (100%)	0 (0%)	N/A	8	1 (100%)
23	**Chen et al. – 2015 [36]**	Case series IV	33 / 19 (57.6%)	50 (28-78)	N/A	Butterfly 23 (69.7%)<br>Unilateral 10 (30.3%)	2-A 11 (33.3%)<br>3-AA 4 (12.1%)<br>4-GBM 18 (54.6%)	PR 20 (60.6%)<br>STR 13 (39.4%)	No 33 (100%)	N/A	N/A	N/A	N/A	17 (51.5%)	8 (0.5-24)	14 (0.1-60)	8 (24.2%)
24	**Burks et al. – 2016 [37]**	Case series IV	40 / 29 (72.5%)	49 (24-77)	Genu/Body 40 (100%)	Butterfly 40 (100%)	2-A 13 (32.5%)<br>3-AA 6 (15%)<br>4-GBM 21 (52.5%)	GTR 33 (82.5%)<br>STR 7 (17.5%)	Yes 40 (100%)	Transient 4 (10%)<br>Persistent 20 (50%)	N/A	N/A	N/A	N/A	N/A	15 (5-18)	30 (75%)
25	**Hu et al. – 2016 [38]**	Case report V	1 / 0 (0%)	44	Genu/Body	Butterfly	4-GBM	GTR	Yes	Persistent	0 (0%)	0 (0%)	1 (100%)	1 (100%)	3	7	1 (100%)
26	**Yapıcı-Eser et al. – 2016 [39]**	Case report V	1 / 0 (0%)	46	Body/Splenium	Butterfly	4-GBM	PR	Yes	0 (0%)	0 (0%)	0 (0%)	1 (100%)	0 (0%)	N/A	0.5	1 (100%)
27	**Krishnan et al. – 2017 [40]**	Case report V	1 / 1 (100%)	54	Genu	Butterfly	4-GBM	Biopsy	No	0 (0%)	1 (100%)	0 (0%)	0 (0%)	0 (0%)	N/A	3	1 (100%)

28	**Mistry et al. – 2017 [41]**	Case series IV	52 /<br>29 (55.8%)	61<br>(20-82)	N/A	N/A	4-GBM 52 (100%)	STR 30 (57.7%)<br>GTR 22 (42.3%)	Yes 52 (100%)	N/A	52 (100%)	52 (100%)	N/A	9 (17.3%)	4<br>(0.1-32)	7.2<br>(0.1-48.5)	2 (3.8%)
29	**Yang et al. – 2017 [42]**	Case series IV	43 /<br>21 (48.8%)	54<br>(21-67)	N/A	Butterfly 43 (100%)	4-GBM 43 (100%)	STR 41 (95.4%)<br>GTR 2 (4.6%)	No 41 (95.4%)<br>Yes 2 (4.6%)	N/A	21 (48.8%)	35 (81.3%)	N/A	N/A	N/A	10.7<br>(6-17)	2 (4.6%)
30	**Amoroso et al. – 2018 [43]**	Case report V	1 /<br>0 (0%)	74	Genu	Unilateral	4-GBM	GTR	Yes	0 (0%)	1 (100%)	0 (0%)	0 (0%)	1 (100%)	11	24	0 (0%)
31	**Dayani et al. – 2018 [44]**	Case series IV	39 /<br>23 (59%)	55<br>(20-83)	Genu 19 (48.7%)<br>Splenium 11 (28.3%)<br>Body 5 (12.8%)<br>Genu/Body 4 (10.3%)	Butterfly 39 (100%)	4-GBM 39 (100%)	Biopsy 25 (64.1%)<br>STR 9 (23.1%)<br>PR 3 (7.7%)<br>GTR 2 (5.1%)	No 27 (69.2%)<br>Yes 12 (30.8%)	Transient 4 (28.6%)<br>Persistent 1 (7.1%)	22 (56.4%)	17 (43.6%)	2 (5.1%)	N/A	N/A	3.2<br>(0.3-25.9)	0 (0%)
32	**Opoku-Darko et al. – 2018 [12]**	Case series IV	29 /<br>18 (62.1%)	60<br>(45-78)	Genu/Body 15 (51.7%)<br>Body/Splenium 14 (48.3%)	Butterfly 29 (100%)	4-GBM 29 (100%)	Biopsy 20 (69%)<br>GTR 5 (17.2%)<br>PR 4 (13.8%)	No 24 (82.7%)<br>Yes 5 (17.3%)	Persistent 3 (10.3%)	19 (65.5%)	19 (65.5%)	N/A	N/A	N/A	3.3<br>(0.1-24)	3 (10.3%)
33	**Azriel et al. – 2019 [45]**	Case report V	1 /<br>1 (100%)	58	Body	Corpus callosum only	4-GBM	Biopsy	No	0 (0%)	0 (0%)	0 (0%)	0 (0%)	0 (0%)	N/A	1	0 (0%)
34	**Esfahani et al. – 2019 [46]**	Case report V	1 /<br>0 (0%)	9	Body	Unilateral	4-GBM	GTR	Yes	0 (0%)	0 (0%)	0 (0%)	0 (0%)	0 (0%)	N/A	1	0 (0%)
35	**La Rocca et al. – 2019 [47]**	Case report V	1 /<br>0 (0%)	48	Splenium	Butterfly	4-GBM	Biopsy	No	0 (0%)	1 (100%)	1 (100%)	0 (0%)	0 (0%)	N/A	3	0 (0%)
36	**Sander et al. – 2019 [48]**	Case report V	1 /<br>0 (0%)	63	Splenium	Butterfly	4-GBM	PR	No	Persistent	1 (100%)	1 (100%)	1 (100%)	1 (100%)	12	16	0 (0%)
37	**Werner et al. – 2019 [49]**	Case report V	1 /<br>1 (100%)	60	Body	Butterfly	4-GBM	Biopsy	No	0 (0%)	1 (100%)	1 (100%)	0 (0%)	1 (100%)	10	11	0 (0%)
38	**Bouali et al. – 2020 [50]**	Case report V	1 /<br>1 (100%)	0.5	Genu/Body	Unilateral	4-GS	GTR	Yes	0 (0%)	0 (0%)	0 (0%)	1 (100%)	1 (100%)	17	17	0 (0%)
39	**De Macedo Filho et al. – 2020 [51]**	Case report V	1 /<br>1 (100%)	44	Body/Splenium	Butterfly	4-GS	STR	No	Persistent	1 (100%)	1 (100%)	0 (0%)	1 (100%)	1	1.5	0 (0%)
40	**Finneran et al. – 2020 [52]**	Case report V	1 /<br>0 (0%)	44	Genu/Body	Butterfly	4-GBM	Biopsy	No	0 (0%)	1 (100%)	1 (100%)	1 (100%)	1 (100%)	36	60	0 (0%)
41	**Forster et al. – 2020 [53]**	Case series IV	21 /<br>9 (%)	41<br>(24-76)	N/A	Unilateral 13 (61.9%)<br>Butterfly 8 (38.1%)	2-ODG 2 (9.5%)<br>2-A 1 (4.8%)<br>4-GBM 17 (81%)	GTR 15 (71.4%)<br>STR 6 (28.6%)	Yes 21 (100%)	Transient 6 (28.6%)<br>Persistent 2 (9.5%)	18 (85.7%)	17 (81%)	4 (19%)	3 (14.3%)	12.5<br>(7.6-19)	12.6<br>(2-30.9)	12 (57.1%)
42	**Jamshidi et al. – 2020 [54]**	Case series IV	3 /<br>2 (66.7%)	61<br>(59-73)	Splenium 2 (66.7%)<br>Genu 1 (33.3%)	Butterfly 3 (100%)	4-GBM 3 (100%)	Biopsy 3 (100%)	No 3 (100%)	0 (0%)	3 (100%)	3 (100%)	3 (100%)	0 (0%)	N/A	8<br>(1-8)	3 (100%)
43	**McKinnon et al. – 2020 [55]**	Case series IV	2 /<br>0 (0%)	46.5<br>(33-60)	Splenium 2 (100%)	Butterfly 1 (50%)<br>Unilateral 1 (50%)	1-GG 1 (50%)<br>4-GBM 1 (50%)	STR 2 (100%)	Yes 2 (100%)	Transient 1 (50%)<br>Persistent 1 (50%)	1 (50%)	1 (50%)	2 (100%)	0 (0%)	N/A	3	2 (100%)
44	**Sato et al. – 2020 [56]**	Case report V	1 /<br>1 (100%)	77	Genu/Body	Unilateral	4-GBM	GTR	Yes	Permanent	1 (100%)	1 (100%)	1 (100%)	1 (100%)	7	15	0 (0%)
45	**Alshoabi et al. – 2021 [57]**	Case report V	1 /<br>1 (100%)	48	Body/Splenium	Butterfly	2-A	Biopsy	No	0 (0%)	0 (0%)	0 (0%)	0 (0%)	0 (0%)	N/A	0.1	0 (0%)
46	**Boaro et al. – 2021 [13]**	Case series IV	62 /<br>32 (%)	64<br>(45-76)	Genu 26 (41.9%)<br>Splenium 22 (35.5%)<br>Body 14 (22.6%)	Butterfly 62 (100%)	4-GBM 62 (100%)	Biopsy 36 (58.1%)<br>STR 23 (37.1%)<br>GTR 3 (4.8%)	No 36 (58.1%)<br>Yes 26 (41.9%)	Transient 5 (8.1%)<br>Persistent 14 (22.6%)	47 (75.8%)	44 (71%)	N/A	35 (56.5%)	5.9<br>(2-9)	8.7<br>(0.5-26)	15 (24.2%)
47	**Cui et al. – 2021 [14]**	Case series IV	77 /<br>43 (%)	50<br>(38-78)	Genu 46 (59.7%)<br>Genu/Body 11 (14.3%)<br>Splenium 10 (13%)<br>Body 7 (9.1%)<br>Body/Splenium 3 (3.9%)	Butterfly 77 (100%)	4-GBM 77 (100%)	GTR 41 (53.2%)<br>STR 23 (29.9%)<br>PR 13 (16.9%)	Yes 77 (100%)	N/A	57 (74%)	77 (100%)	N/A	51 (66.2%)	10<br>(0.3-51)	15<br>(0.1-52)	17 (22.1%)
48	**Franco et al. – 2021 [58]**	Case series IV	55 /<br>34 (%)	64<br>(48-81)	Genu 27 (49%)<br>Body 11 (20%)<br>Body/Splenium 6 (11%)<br>Splenium 6 (11%)<br>Genu/Body 5 (9%)	N/A	4-GBM 55 (100%)	Biopsy 30 (54.5%)<br>STR 17 (30.9%)<br>GTR 8 (14.5%)	No 30 (54.5%)<br>Yes 25 (45.5%)	Persistent 13 (20.3%)	19 (34.5%)	29 (52.7%)	18 (32.7%)	N/A	N/A	8.4<br>(0.1-43.3)	10 (18.2%)
49	**Kayahara et al. – 2021 [59]**	Case report V	1 /<br>0 (0%)	22	Splenium	Unilateral	1-PA	GTR	Yes	Transient	0 (0%)	0 (0%)	1 (100%)	0 (0%)	N/A	36	1 (100%)
50	**Shibahara et al. – 2021 [60]**	Case series IV	8 /<br>4 (50%)	56<br>(27-69)	Splenium 8 (100%)	Unilateral 8 (100%)	2-A 2 (25%)<br>4-GBM 6 (75%)	GTR 5 (62.5%)<br>PR 2 (25%)<br>STR 1 (12.5%)	Yes 8 (100%)	N/A	N/A	N/A	N/A	N/A	N/A	6<br>(1-12)	8 (100%)
51	**Dadario et al. – 2022 [62]**	Case series IV	70 /<br>43 (%)	49<br>(18-73)	Genu 47 (78%)<br>Splenium 19 (27%)<br>Body 4 (6%)	Butterfly 70 (100%)	2-A 4 (5.7%)<br>3-AODG 3 (4.3%)<br>3-AA 2 (2.9%)<br>4-GBM 60 (85.7%)<br>4-GS 1 (1.4%)	GTR 63 (90%)<br>STR 7 (10%)	Yes 70 (100%)	Persistent 21 (30%)	70 (100%)	70 (100%)	N/A	N/A	N/A	14<br>(1-53)	12 (17.1%)
52	**Sharma et al. – 2022 [63]**	Case report V	1 /<br>1 (100%)	60	Genu/Body	Butterfly	4-GBM	Biopsy	No	0 (0%)	0 (0%)	0 (0%)	0 (0%)	0 (0%)	N/A	0.1	0 (0%)

Abbreviations: **A**, Astrocytoma; **AA**, Anaplastic astrocytoma; **AOA**, Anaplastic oligoastrocytoma; **AODG**, Anaplastic oligodendroglioma; **GBM**, Glioblastoma; **GG**, Ganglioglioma; **GS**, Gliosarcoma; **GTR**, Gross-total resection (90-100%); **ODG**, oligodendroglioma; **PA**, Pilocytic astrocytoma; **PR**, Partial resection (<80%); **SE**, Subependymoma; **STR**, Subtotal resection (80-90%).
