# Supplementary material for: Gliomas Infiltrating the Corpus Callosum: A Systematic Review of the Literature
Source: Cancers (Basel). 2022 May 19;14(10):2507. doi: 10.3390/cancers14102507 (PMC9139932; doi:10.3390/cancers14102507)
Supplement: Supplementary file 1 [file cancers-14-02507-s001.zip › Supplementary File 2 - Risk of bias assessment for included studies.pdf]

Supplementary File S2. Risk of bias assessments for included studies

**Joanna Briggs Institute Checklist for Case Reports – Criteria**
1. Were patient's demographic characteristics clearly described?
2. Was the patient's history clearly described and presented as a timeline?
3. Was the current clinical condition of the patient on presentation clearly described?
4. Were diagnostic tests or assessment methods and the results clearly described?
5. Was the intervention(s) or treatment procedure(s) clearly described?
6. Was the post-intervention clinical condition clearly described?
7. Were adverse events (harms) or unanticipated events identified and described?
8. Does the case report provide takeaway lessons?
**Responses Options**: Yes, No, Unclear, Not Applicable (NA)
**Quality Rating**: Poor 0 – 2; Fair 3 – 5; Good 6 – 8

Study	1	2	3	4	5	6	7	8	Rating
Cabot et al. – 1949[61]	Yes	Yes	Yes	Yes	Yes	Yes	Yes	No	7 – Good
Anderson et al. – 1970[64]	Yes	Yes	Yes	Yes	Yes	Yes	Yes	Yes	8 – Good
Zant et al. – 1982[65]	Yes	Yes	Yes	Yes	Yes	Yes	Yes	Yes	8 – Good
Kato et al. – 1990[66]	Yes	Yes	Yes	Yes	Yes	Yes	Yes	Yes	8 – Good
Lindboe et al. – 1992[67]	Yes	Yes	Yes	Yes	Yes	Yes	Yes	Yes	8 – Good
Ideguchi et al. – 1998[22]	Yes	Yes	Yes	Yes	Yes	Yes	Yes	Yes	8 – Good
Bampoe et al. – 1999[23]	Yes	Yes	Yes	Yes	Yes	Yes	Yes	Yes	8 – Good
Price et al. – 2004[24]	Yes	Yes	Yes	Yes	Yes	Yes	Yes	Yes	8 – Good
Pallud et al. – 2005[25]	Yes	Yes	Yes	Yes	Yes	Yes	Yes	Yes	8 – Good
Voloschin et al. – 2005[26]	Yes	Yes	Yes	Yes	Yes	Yes	Yes	Yes	8 – Good
Zakrewska et al. – 2007[27]	Yes	Yes	Yes	Yes	Yes	Yes	Yes	Yes	8 – Good
Tsutsumi et al. – 2008[28]	Yes	Yes	Yes	Yes	Yes	Yes	Yes	Yes	8 – Good
Agrawal et al. – 2009[29]	Yes	Yes	Yes	Yes	Yes	Yes	Yes	Yes	8 – Good
Kumar et al. – 2011[31]	Yes	Yes	Yes	Yes	Yes	Yes	Yes	Yes	8 – Good
Monaco et al. – 2011[32]	Yes	Yes	Yes	Yes	Yes	Yes	Yes	Yes	8 – Good
Witoonpanich et al. – 2011[33]	Yes	Yes	Yes	Yes	Yes	Yes	Yes	Yes	8 – Good
Lee et al. – 2014[35]	Yes	Yes	Yes	Yes	Yes	Yes	Yes	Yes	8 – Good
Hu et al. – 2016[38]	Yes	Yes	Yes	Yes	Yes	Yes	Yes	Yes	8 – Good
Yapıcı-Eser et al. – 2016[39]	Yes	Yes	Yes	Yes	Yes	Yes	Yes	Yes	8 – Good
Krishnan et al. – 2017[40]	Yes	Yes	Yes	Yes	Yes	Yes	Yes	Yes	8 – Good
Amoroso et al. – 2018[43]	Yes	Yes	Yes	Yes	Yes	Yes	Yes	Yes	8 – Good
Azriel et al. – 2019[45]	Yes	Yes	Yes	Yes	Yes	Yes	Yes	Yes	8 – Good
Esfahani et al. – 2019[46]	Yes	Yes	Yes	Yes	Yes	Yes	Yes	Yes	8 – Good
La Rocca et al. – 2019[47]	Yes	Yes	Yes	Yes	Yes	Yes	Yes	Yes	8 – Good
Sander et al. – 2019[48]	Yes	Yes	Yes	Yes	Yes	Yes	Yes	Yes	8 – Good

Werner et al. – 2019[49]	Yes	Yes	Yes	Yes	Yes	Yes	Yes	No	7 – Good
Bouali et al. – 2020[50]	Yes	Yes	Yes	Yes	Yes	Yes	Yes	Yes	8 – Good
De Macedo Filho et al. – 2020[51]	Yes	Yes	Yes	Yes	Yes	Yes	Yes	Yes	8 – Good
Finneran et al. – 2020[52]	Yes	Yes	Yes	Yes	Yes	Yes	Yes	Yes	8 – Good
Sato et al. – 2020[56]	Yes	Yes	Yes	Yes	Yes	Yes	Yes	Yes	8 – Good
Alshoabi et al. – 2021[57]	Yes	Yes	Yes	Yes	Yes	Yes	Yes	Yes	8 – Good
Kayahara et al. – 2021[59]	Yes	Yes	Yes	Yes	Yes	Yes	Yes	Yes	8 – Good
Sharma et al. – 2022[63]	Yes	Yes	Yes	Yes	Yes	Yes	Yes	Yes	8 – Good

Joanna Briggs Institute Checklist for Case Series – Criteria
1. Were there clear criteria for inclusion in the case series?
2. Was the condition measured in a standard, reliable way for all participants included in the case series?
3. Were valid methods used for identification of the condition for all participants included in the case series?
4. Did the case series have consecutive inclusion of participants?
5. Did the case series have complete inclusion of participants?
6. Was there clear reporting of the demographics of the participants in the study?
7. Was there clear reporting of clinical information of the participants?
8. Were the outcomes or follow up results of cases clearly reported?
9. Was there clear reporting of the presenting site(s)/clinic(s) demographic information?
10. Was statistical analysis appropriate?
**Responses Options**: Yes, No, Unclear, Not Applicable (NA)
**Quality Rating**: Poor 0 – 3; Fair 4 – 7; Good 8 – 10

Study	1	2	3	4	5	6	7	8	9	10	Rating
Bauman et al. – 1998[21]	Yes	Yes	Yes	Yes	Yes	Yes	Yes	Yes	No	NA	8 – Good
Duffau et al. – 2004[9]	Yes	Yes	Yes	Yes	Yes	Yes	Yes	Yes	No	NA	8 – Good
Steiger et al. – 2009[30]	Yes	Yes	Yes	Yes	Yes	Yes	Yes	Yes	No	NA	8 – Good
Dziurzynski et al. – 2012[34]	Yes	Yes	Yes	Yes	Yes	Yes	Yes	Yes	Yes	Yes	10 – Good
Chaichana et al. – 2014[10]	Yes	Yes	Yes	Yes	Yes	Yes	Yes	Yes	No	Yes	9 – Good
Chen et al. – 2015[36]	Yes	Yes	Yes	Yes	Yes	Yes	Yes	Yes	No	Yes	9 – Good
Burks et al. – 2016[37]	Yes	Yes	Yes	Yes	Yes	Yes	Yes	Yes	Yes	Yes	10 – Good
Mistry et al. – 2017[41]	Yes	Yes	Yes	Yes	Yes	Yes	Yes	Yes	Yes	Yes	10 – Good
Yang et al. – 2017[42]	Yes	Yes	Yes	Yes	Yes	Yes	Yes	Yes	Yes	Yes	10 – Good
Dayani et al. – 2018[44]	Yes	Yes	Yes	Yes	Yes	Yes	Yes	Yes	Yes	Yes	10 – Good
Opoku-Darko et al. – 2018[12]	Yes	Yes	Yes	Yes	Yes	Yes	Yes	Yes	Yes	Yes	10 – Good
Forster et al. – 2020[53]	Yes	Yes	Yes	Yes	Yes	Yes	Yes	Yes	No	NA	8 – Good
Jamshidi et al. – 2020[54]	Yes	Yes	Yes	Yes	Yes	Yes	Yes	Yes	No	NA	8 – Good
McKinnon et al. – 2020[55]	Yes	Yes	Yes	Yes	Yes	Yes	Yes	Yes	No	NA	8 – Good
Boaro et al. – 2021[13]	Yes	Yes	Yes	Yes	Yes	Yes	Yes	Yes	Yes	Yes	10 – Good
Cui et al. – 2021[14]	Yes	Yes	Yes	Yes	Yes	Yes	Yes	Yes	No	Yes	9 – Good
Franco et al. – 2021[58]	Yes	Yes	Yes	Yes	Yes	Yes	Yes	Yes	Yes	Yes	10 – Good
Shibahara et al. – 2021[60]	Yes	Yes	Yes	Yes	Yes	Yes	Yes	Yes	Yes	NA	9 – Good
Dadario et al. – 2022[62]	Yes	Yes	Yes	Yes	Yes	Yes	Yes	Yes	No	NA	8 – Good
